# Supplementary material for: Evolution of developmental sequences in lepidosaurs
Source: PeerJ. 2017 Apr 27;5:e3262. doi: 10.7717/peerj.3262 (PMC5410152; doi:10.7717/peerj.3262)
Supplement: Table S1 — Molecular phylogeny, ordered characters. [file peerj-05-3262-s007.docx]

| **Character** | **Character state transformation** | **Relative change** | **Row** | **Column** |
| --- | --- | --- | --- | --- |
| Unidentata |  |  |  |  |
| 9 | 0→1 | 1 | 5 | 3 |
| 13 | 0→1/2 | 1,5 | 6 | 3 |
| Scincoidea (*Mabuya*) |  |  |  |  |
| 15 | 1→2 | 1 | 6 | 5 |
| 19 | 2→0 | -2 | 7 | 4 |
| 40 | 2→1 | -1 | 10 | 4 |
| 42 | 2→1 | -1 | 10 | 6 |
| 44 | 2→0 | -2 | 10 | 8 |
| 45 | 1→0 | -1 | 10 | 9 |
| 136 | 1→2 | 1 | 17 | 16 |
| Gymnophthalmidae |  |  |  |  |
| 102 | 2→1 | -1 | 15 | 11 |
| 104 | 1/2→0 | -1,5 | 15 | 13 |
| 133 | 2→1 | -1 | 17 | 13 |
| 134 | 2→1 | -1 | 17 | 14 |
| 171 | 2→1 | -1 | 19 | 18 |
| Toxicofera |  |  |  |  |
| 9 | 1→2 | 1 | 5 | 2 |
| 20 | 2→1 | -1 | 7 | 5 |
| Serpentes |  |  |  |  |
| 133 | 2→0/1 | -1,5 | 17 | 13 |
| 136 | 1→0 | -1 | 17 | 16 |
| *Thamnophis* + *Vipera* |  |  |  |  |
| 104 | 0/1→2 | 1,5 | 15 | 13 |
| 120 | 2→1 | -1 | 16 | 15 |
| *Varanus rosenbergi* + *V. panoptes* |  |  |  |  |
| 134 | 2→1 | -1 | 17 | 14 |
| Iguania |  |  |  |  |
| 36 | 1→2 | -1 | 9 | 8 |
| Chamaeleonidae |  |  |  |  |
| 45 | 1→2 | 1 | 10 | 9 |
| *Agama* + *Calotes* |  |  |  |  |
| 104 | 1→2 | 1 | 15 | 13 |
| 105 | 1→2 | 1 | 15 | 14 |
| 135 | 2→1 | -1 | 17 | 15 |
| Pleurodonta excl. *Tropidurus* |  |  |  |  |
| 134 | 2→1 | -1 | 17 | 14 |
| *Liolaemus* |  |  |  |  |
| 135 | 2→0 | -2 | 17 | 15 |
| *Strophurus* |  |  |  |  |
| 104 | 2→1 | -1 | 15 | 13 |
| 105 | 1/2→0 | -1,5 | 15 | 14 |
| *Amalosia* |  |  |  |  |
| 91 | 2→1 | -1 | 14 | 13 |
| 135 | 2→1 | -1 | 17 | 15 |
| 136 | 1→2 | 1 | 17 | 16 |
| 151 | 2→1 | -1 | 18 | 15 |
| 153 | 2→1 | -1 | 18 | 17 |
| *Eublepharis* |  |  |  |  |
| 91 | 2→1 | -1 | 14 | 13 |
| 133 | 2→1 | -1 | 17 | 13 |
| 135 | 2→0 | -2 | 17 | 15 |
| *Gehyra* |  |  |  |  |
| 55 | 2→1 | -1 | 11 | 10 |
| 65 | 2→1 | -1 | 12 | 10 |
| 104 | 2→0 | -2 | 15 | 13 |
| 136 | 1→0 | -1 | 17 | 16 |
| *Chondrodactylus* |  |  |  |  |
| 119 | 1/2→0 | -1,5 | 16 | 14 |
| 134 | 0/1→2 | 1,5 | 17 | 14 |
| 136 | 1→2 | 1 | 17 | 16 |
| 153 | 2→1 | -1 | 18 | 17 |
| *Tarentola* |  |  |  |  |
| 54 | 2→1 | -1 | 11 | 9 |
| 66 | 1→2 | 1 | 12 | 11 |
| 103 | 2→0 | -2 | 15 | 12 |
| 132 | 2→1 | -1 | 17 | 12 |
| 150 | 2→0 | -2 | 18 | 14 |
| *Zootoca* |  |  |  |  |
| 105 | 0/1→2 | 1,5 | 15 | 14 |
| 120 | 2→1 | -1 | 16 | 15 |
| 136 | 1→2 | 1 | 17 | 16 |
| 153 | 2→1 | -1 | 18 | 17 |
| *Calyptommatus* |  |  |  |  |
| 103 | 2→1 | -1 | 15 | 12 |
| 133 | 1→0 | -1 | 17 | 13 |
| 134 | 1→0 | -1 | 17 | 14 |
| *Nothobachia* |  |  |  |  |
| 66 | 1→0 | -1 | 12 | 11 |
| 91 | 2→1 | -1 | 14 | 13 |
| *Python* |  |  |  |  |
| 169 | 2→1 | -1 | 19 | 16 |
| 187 | 1/2→0 | -1,5 | 20 | 16 |
| *Boaedon* |  |  |  |  |
| 53 | 2→1 | -1 | 11 | 8 |
| 99 | 2→1 | -1 | 15 | 8 |
| 102 | 2→1 | -1 | 15 | 11 |
| 118 | 2→1 | -1 | 16 | 13 |
| *Thamnophis* |  |  |  |  |
| 3 | 1/2→0 | -1,5 | 3 | 2 |
| 5 | 2→0 | -2 | 4 | 2 |
| 14 | 1→2 | 1 | 6 | 4 |
| 17 | 2→1 | -1 | 7 | 2 |
| 20 | 1→0 | -1 | 7 | 5 |
| 44 | 2→1 | -1 | 10 | 8 |
| 133 | 2→1 | -1 | 17 | 13 |
| 135 | 2→0 | -2 | 17 | 15 |
| 136 | 1/2→0 | -1,5 | 17 | 16 |
| *Vipera* |  |  |  |  |
| 6 | 1→0 | -1 | 4 | 3 |
| 13 | 1/2→0 | -1,5 | 6 | 3 |
| 133 | 1→2 | 1 | 17 | 13 |
| 136 | 0→2 | 2 | 17 | 16 |
| *Varanus rosenbergi* |  |  |  |  |
| 105 | 1→2 | 1 | 15 | 14 |
| 118 | 2→1 | -1 | 16 | 13 |
| 119 | 2→0 | -2 | 16 | 14 |
| 120 | 2→0 | -2 | 16 | 15 |
| 136 | 1→0 | -1 | 17 | 16 |
| 150 | 2→1 | -1 | 18 | 14 |
| *Varanus panoptes* |  |  |  |  |
| 91 | 1/2→0 | -1,5 | 14 | 13 |
| 99 | 2→0 | -2 | 15 | 8 |
| 102 | 2→1 | -1 | 15 | 11 |
| 104 | 1→0 | -1 | 15 | 13 |
| 133 | 2→0 | -2 | 17 | 13 |
| 136 | 1→0 | -1 | 17 | 16 |
| 171 | 2→1 | -1 | 19 | 18 |
| *Varanus indicus* |  |  |  |  |
| 103 | 2→0 | -2 | 15 | 12 |
| 152 | 2→1 | -1 | 18 | 16 |
| *Pogona* |  |  |  |  |
| 66 | 2→1 | -1 | 12 | 11 |
| 118 | 2→1 | -1 | 16 | 13 |
| 119 | 2→1 | -1 | 16 | 14 |
| 136 | 1→2 | 1 | 17 | 16 |
| 153 | 2→1 | -1 | 18 | 17 |
| *Calotes* |  |  |  |  |
| 10 | 1→0 | -1 | 5 | 4 |
| 19 | 1→0 | -1 | 7 | 4 |
| 20 | 1→0 | -1 | 7 | 5 |
| 44 | 2→1 | -1 | 10 | 8 |
| 45 | 1→0 | -1 | 10 | 9 |
| 54 | 2→1 | -1 | 11 | 9 |
| 136 | 1→0 | -1 | 17 | 16 |
| 152 | 2→1 | -1 | 18 | 16 |
| *Agama* |  |  |  |  |
| 13 | 2→1 | -1 | 6 | 3 |
| 15 | 1→0 | -1 | 6 | 5 |
| 120 | 1/2→0 | -1,5 | 16 | 15 |
| 135 | 1→0 | -1 | 17 | 15 |
| *Furcifer* |  |  |  |  |
| 10 | 1→0 | -1 | 5 | 4 |
| 20 | 1→2 | 1 | 7 | 5 |
| 25 | 2→1 | -1 | 8 | 4 |
| 28 | 2→1 | -1 | 8 | 7 |
| 32 | 2→1 | -1 | 9 | 4 |
| 35 | 2→1 | -1 | 9 | 7 |
| 36 | 2→1 | -1 | 9 | 8 |
| 55 | 2→1 | -1 | 11 | 10 |
| 104 | 1→2 | 1 | 15 | 13 |
| 105 | 1→2 | 1 | 15 | 14 |
| 152 | 2→1 | -1 | 18 | 16 |
| 153 | 2→1 | -1 | 18 | 17 |
| *Chamaeleo* |  |  |  |  |
| 3 | 1/2→0 | -1,5 | 3 | 2 |
| 5 | 2→0 | -2 | 4 | 2 |
| 6 | 2→1 | -1 | 4 | 3 |
| 8 | 2→0 | -2 | 5 | 2 |
| 9 | 2→1 | -1 | 5 | 3 |
| 12 | 2→1 | -1 | 6 | 2 |
| 14 | 0/1→2 | 1,5 | 6 | 4 |
| 15 | 1→2 | 1 | 6 | 5 |
| 17 | 2→0 | -2 | 7 | 2 |
| 18 | 2→1 | -1 | 7 | 3 |
| 23 | 2→1 | -1 | 8 | 2 |
| 27 | 2→1 | -1 | 8 | 6 |
| 104 | 2→1 | -1 | 15 | 13 |
| *Tropidurus* |  |  |  |  |
| 34 | 2→0 | -2 | 9 | 6 |
| 51 | 2→0 | -2 | 11 | 6 |
| 104 | 1→0 | -1 | 15 | 13 |
| *Iguana* |  |  |  |  |
| 91 | 1/2→0 | -1,5 | 14 | 13 |
| 149 | 2→0 | -2 | 18 | 13 |
| 152 | 2→0 | -2 | 18 | 16 |
| *Uta* |  |  |  |  |
| 15 | 1→0 | -1 | 6 | 5 |
| 19 | 1/2→0 | -1,5 | 7 | 4 |
| 20 | 1→0 | -1 | 7 | 5 |
| 26 | 2→1 | -1 | 8 | 5 |
| *Anolis* |  |  |  |  |
| 102 | 2→1 | -1 | 15 | 11 |
| 103 | 2→0 | -2 | 15 | 12 |
| *L. gravenhorsti* |  |  |  |  |
| 25 | 1→0 | -1 | 8 | 4 |
| 54 | 2→0 | -2 | 11 | 9 |
| 55 | 2→0 | -2 | 11 | 10 |
| 104 | 1→2 | 1 | 15 | 13 |
| 120 | 2→1 | -1 | 16 | 15 |
| 150 | 2→1 | -1 | 18 | 14 |
| 151 | 2→1 | -1 | 18 | 15 |
| 152 | 2→1 | -1 | 18 | 16 |
| *L. tenuis* |  |  |  |  |
| 5 | 2→1 | -1 | 4 | 2 |
| 14 | 1→2 | 1 | 6 | 4 |
| 132 | 2→1 | -1 | 17 | 12 |
| 190 | 2→1 | -1 | 20 | 19 |
