# Supplementary material for: Evolution of developmental sequences in lepidosaurs
Source: PeerJ. 2017 Apr 27;5:e3262. doi: 10.7717/peerj.3262 (PMC5410152; doi:10.7717/peerj.3262)
Supplement: Table S4 [file peerj-05-3262-s010.docx]

| **Event** | **As row** | **As column** | **TRC** | **TAC** |
| --- | --- | --- | --- | --- |
| Unidentata |  |  |  |  |
| 3 | 0 | 2 | -2 | 2 |
| 5 | 1 | 0 | 1 | 1 |
| 6 | 1 | 0 | 1 | 1 |
| Scincoidea (*Mabuya*) |  |  |  |  |
| 4 | 0 | -2 | 2 | 2 |
| 5 | 0 | 1 | -1 | 1 |
| 6 | 1 | -1 | 2 | 2 |
| 7 | -1 | 0 | -1 | 1 |
| 8 | 0 | -1 | 1 | 1 |
| 10 | -3 | 0 | -3 | 3 |
| Gymnophthalmidae |  |  |  |  |
| 11 | 0 | -1 | 1 | 1 |
| 13 | 0 | -1 | 1 | 1 |
| 15 | -2 | 0 | -2 | 2 |
| 18 | 0 | -1 | 1 | 1 |
| 19 | -1 | 0 | -1 | 1 |
| *Liolaemus* |  |  |  |  |
| 14 | 0 | 1 | -1 | 1 |
| 15 | 1 | -1 | 2 | 2 |
| 17 | -1 | 0 | -1 | 1 |
| *Amalosia* |  |  |  |  |
| 13 | 0 | -1 | 1 | 1 |
| 14 | -1 | -1 | 0 | 2 |
| 15 | 0 | -2 | 2 | 2 |
| 16 | -1 | 0 | -1 | 1 |
| 17 | -1 | -1 | 0 | 2 |
| 18 | -2 | 0 | -2 | 2 |
| *Eublepharis* |  |  |  |  |
| 13 | 0 | -2 | 2 | 2 |
| 14 | -1 | -1 | 0 | 2 |
| 15 | 0 | -1 | 1 | 1 |
| 16 | -1 | -1 | 0 | 2 |
| 17 | -3 | 0 | -3 | 3 |
| *Gehyra* |  |  |  |  |
| 10 | 0 | -2 | 2 | 2 |
| 11 | -1 | 0 | -1 | 1 |
| 12 | -1 | 0 | -1 | 1 |
| 13 | 0 | -1 | 1 | 1 |
| 15 | -1 | 0 | -1 | 1 |
| 16 | 0 | -1 | 1 | 1 |
| 17 | -1 | 0 | -1 | 1 |
| *Tarentola* |  |  |  |  |
| 9 | 0 | -1 | 1 | 1 |
| 11 | -1 | 1 | -2 | 2 |
| 12 | 1 | -2 | 3 | 3 |
| 14 | 0 | -1 | 1 | 1 |
| 15 | -1 | 0 | -1 | 1 |
| 17 | -1 | 0 | -1 | 1 |
| 18 | -1 | 0 | -1 | 1 |
| *Python* |  |  |  |  |
| 16 | 0 | -2 | 2 | 2 |
| 19 | -1 | 0 | -1 | 1 |
| 20 | -1 | 0 | -1 | 1 |
| *Boaedon* |  |  |  |  |
| 8 | 0 | -2 | 2 | 2 |
| 11 | -1 | -1 | 0 | 2 |
| 14 | 0 | -1 | 1 | 1 |
| 15 | -2 | 0 | -2 | 2 |
| 16 | -1 | -1 | 0 | 2 |
| 20 | -1 | 0 | -1 | 1 |
| *Thamnophis* |  |  |  |  |
| 2 | 0 | -3 | 3 | 3 |
| 3 | -1 | 0 | -1 | 1 |
| 4 | -1 | 1 | -2 | 2 |
| 6 | 1 | 0 | 1 | 1 |
| 7 | -1 | 0 | -1 | 1 |
| 8 | 0 | -1 | 1 | 1 |
| 10 | -1 | 0 | -1 | 1 |
| 13 | 0 | -1 | 1 | 1 |
| 15 | 0 | -1 | 1 | 1 |
| 16 | 0 | -1 | 1 | 1 |
| 17 | -3 | 0 | -3 | 3 |
| *Varanus indicus* |  |  |  |  |
| 12 | 0 | -1 | 1 | 1 |
| 15 | -1 | 0 | -1 | 1 |
| 16 | 0 | 0 | 0 | 2 |
| 17 | 1 | 0 | 1 | 1 |
| 18 | -1 | 0 | -1 | 1 |
| *Varanus panoptes* |  |  |  |  |
| 8 | 0 | -1 | 1 | 1 |
| 11 | 0 | -1 | 1 | 1 |
| 13 | 0 | -3 | 3 | 3 |
| 14 | -1 | 0 | -1 | 1 |
| 15 | -3 | 0 | -3 | 3 |
| 17 | -1 | 0 | -1 | 1 |
| 18 | 0 | -1 | 1 | 1 |
| 19 | -1 | 0 | -1 | 1 |
| *Varanus rosenbergi* |  |  |  |  |
| 13 | 0 | -1 | 1 | 1 |
| 14 | 0 | -2 | 2 | 2 |
| 15 | 0 | -1 | 1 | 1 |
| 16 | -3 | 1 | -4 | 4 |
| 17 | 1 | 0 | 1 | 1 |
| 18 | -1 | 0 | -1 | 1 |
| *Pogona* |  |  |  |  |
| 11 | 0 | -1 | 1 | 1 |
| 12 | -1 | 0 | -1 | 1 |
| 13 | 0 | -2 | 2 | 2 |
| 14 | 0 | -1 | 1 | 1 |
| 15 | -1 | -1 | 0 | 2 |
| 16 | -3 | 1 | -4 | 4 |
| 17 | 1 | -1 | 2 | 2 |
| 18 | -1 | 0 | -1 | 1 |
| *Furcifer* |  |  |  |  |
| 4 | 0 | -2 | 2 | 2 |
| 7 | 0 | -2 | 2 | 2 |
| 8 | -2 | -1 | -1 | 3 |
| 9 | -3 | 0 | -3 | 3 |
| 10 | 0 | -1 | 1 | 1 |
| 11 | -1 | 0 | -1 | 1 |
| 16 | 0 | -1 | 1 | 1 |
| 17 | 0 | -1 | 1 | 1 |
| 18 | -2 | 0 | -2 | 2 |
| *Chamaeleo* |  |  |  |  |
| 2 | 0 | -6 | 6 | 6 |
| 3 | -1 | -3 | 2 | 4 |
| 4 | -2 | 1 | -3 | 3 |
| 5 | -2 | 0 | -2 | 2 |
| 6 | 0 | -1 | 1 | 3 |
| 7 | -2 | 0 | -2 | 2 |
| 8 | -2 | 0 | -2 | 2 |
| 13 | 0 | -1 | 1 | 1 |
| 15 | -1 | 0 | -1 | 1 |
| *Tropidurus* |  |  |  |  |
| 6 | 0 | -2 | 2 | 2 |
| 9 | -1 | 0 | -1 | 1 |
| 11 | -1 | 0 | -1 | 1 |
| 12 | 0 | -1 | 1 | 1 |
| 13 | 0 | -1 | 1 | 1 |
| 15 | -2 | 0 | -2 | 2 |
| *Iguana* |  |  |  |  |
| 13 | 0 | -2 | 2 | 2 |
| 14 | -1 | 0 | -1 | 1 |
| 16 | 0 | -1 | 1 | 1 |
| 18 | -2 | 0 | -2 | 2 |
| *Anolis* |  |  |  |  |
| 11 | 0 | -1 | 1 | 1 |
| 12 | 0 | -1 | 1 | 1 |
| 15 | -2 | 0 | -2 | 2 |
| *L. gravenhorsti* |  |  |  |  |
| 4 | 0 | -1 | 1 | 1 |
| 8 | -1 | 0 | -1 | 1 |
| 9 | 0 | -1 | 1 | 1 |
| 10 | 0 | -1 | 1 | 1 |
| 11 | -2 | 0 | -2 | 2 |
| 14 | 0 | -1 | 1 | 1 |
| 15 | 0 | -2 | 2 | 2 |
| 16 | -1 | -1 | 0 | 2 |
| 18 | -3 | 0 | -3 | 3 |
| *L. tenuis* |  |  |  |  |
| 2 | 0 | -1 | 1 | 1 |
| 4 | -1 | 0 | -1 | 1 |
| 12 | 0 | -1 | 1 | 1 |
| 13 | 0 | -2 | 2 | 2 |
| 14 | -1 | 0 | -1 | 1 |
| 15 | -1 | 0 | -1 | 1 |
| 17 | -1 | 0 | -1 | 1 |
| 19 | 0 | -1 | 1 | 1 |
| 20 | -1 | 0 | -1 | 1 |
|  |  |  |  |  |
