# Supplementary material for: Evolution of developmental sequences in lepidosaurs
Source: PeerJ. 2017 Apr 27;5:e3262. doi: 10.7717/peerj.3262 (PMC5410152; doi:10.7717/peerj.3262)
Supplement: Table S5 — Morphological phylogeny, ordered characters. [file peerj-05-3262-s011.docx]

| **Character** | **Character state transformation** | **Relative change** | | **Row** | **Column** | |  |
| --- | --- | --- | --- | --- | --- | --- | --- |
| Iguania |  |  | |  |  | |  |
| 13 | 0/1→2 | 1,5 | | 6 | 3 | |  |
| Pleurodonta |  |  | |  |  | |  |
| 134 | 2→0 | -2 | | 17 | 14 | |  |
| 136 | 1→0 | -1 | | 17 | 16 | |  |
| *Liolaemus* |  |  | |  |  | |  |
| 3 | 2→0 | -2 | | 3 | 2 | |  |
| 8 | 2→0 | -2 | | 5 | 2 | |  |
| 14 | 0→1 | 1 | | 6 | 4 | |  |
| 15 | 1→2 | 1 | | 6 | 5 | |  |
| 23 | 2→1 | -1 | | 8 | 2 | |  |
| 27 | 2→0 | -2 | | 8 | 6 | |  |
| 28 | 1/2→0 | -1,5 | | 8 | 7 | |  |
| 134 | 1→0 | -1 | | 17 | 14 | |  |
| 135 | 2→0 | -2 | | 17 | 15 | |  |
| Chamaeleonidae |  |  | |  |  | |  |
| 45 | 1→2 | 1 | | 10 | 9 | |  |
| *Agama* + *Pogona* |  |  | |  |  | |  |
| 120 | 2→1 | -1 | | 16 | 15 | |  |
| Scleroglossa |  |  | |  |  | |  |
| 6 | 2→1 | -1 | | 4 | 3 | |  |
| 14 | 0→1 | 1 | | 6 | 4 | |  |
| Gekkota except Diplodactylidae |  |  | |  |  | |  |
| 134 | 2→1 | -1 | | 17 | 14 | |  |
| *Varanus panoptes* + *V. rosenbergi* |  |  | |  |  | |  |
| 134 | 2→1 | -1 | | 17 | 14 | |  |
| Serpentes |  |  | |  |  | |  |
| 136 | 1→0 | -1 | | 17 | 16 | |  |
| Scincomorpha |  |  | |  |  | |  |
| 8 | 2→1 | -1 | | 5 | 2 | |  |
| Scincoidea |  |  | |  |  | |  |
| 6 | 1→2 | 1 | | 4 | 3 | |  |
| 10 | 1→0 | -1 | | 5 | 4 | |  |
| 13 | 1→2 | 1 | | 6 | 3 | |  |
| 15 | 1→2 | 1 | | 6 | 5 | |  |
| 19 | 2→0 | -2 | | 7 | 4 | |  |
| 21 | 2→0 | -2 | | 7 | 6 | |  |
| 40 | 2→1 | -1 | | 10 | 4 | |  |
| 42 | 2→1 | -1 | | 10 | 6 | |  |
| 44 | 2→0 | -2 | | 10 | 8 | |  |
| 45 | 1→0 | -1 | | 10 | 9 | |  |
| Gymnophthalmidae |  |  | |  |  | |  |
| 102 | 2→1 | -1 | | 15 | 11 | |  |
| 104 | 2→0 | -2 | | 15 | 13 | |  |
| 105 | 1→0 | -1 | | 15 | 14 | |  |
| 133 | 2→1 | -1 | | 17 | 13 | |  |
| 134 | 2→1 | -1 | | 17 | 14 | |  |
| 171 | 2→1 | -1 | | 19 | 18 | |  |
| *Calotes* | |  | |  |  | |  |
| 19 | | 1→0 | | -1 | 7 | | 4 |
| 20 | | 1→0 | | -1 | 7 | | 5 |
| 44 | | 2→1 | | -1 | 10 | | 8 |
| 45 | | 1→0 | | -1 | 10 | | 9 |
| 54 | | 2→1 | | -1 | 11 | | 9 |
| 136 | | 1→0 | | -1 | 17 | | 16 |
| 152 | | 2→1 | | -1 | 18 | | 16 |
| *Pogona* | |  | |  |  | |  |
| 66 | | 2→1 | | -1 | 12 | | 11 |
| 104 | | 2→1 | | -1 | 15 | | 13 |
| 118 | | 2→1 | | -1 | 16 | | 13 |
| 119 | | 2→1 | | -1 | 16 | | 14 |
| 136 | | 1→2 | | 1 | 17 | | 16 |
| 153 | | 2→1 | | -1 | 18 | | 17 |
| *Agama* | |  | |  |  | |  |
| 120 | | 1→0 | | -1 | 16 | | 15 |
| 135 | | 1/2→0 | | -1,5 | 17 | | 15 |
| *Furcifer* | |  | |  |  | |  |
| 25 | | 2→1 | | -1 | 8 | | 4 |
| 32 | | 2→1 | | -1 | 9 | | 4 |
| 35 | | 2→1 | | -1 | 9 | | 5 |
| 36 | | 2→1 | | -1 | 9 | | 6 |
| 55 | | 2→1 | | -1 | 11 | | 10 |
| 152 | | 2→1 | | -1 | 18 | | 16 |
| 153 | | 2→1 | | -1 | 18 | | 17 |
| *Chamaeleo* | |  | |  |  | |  |
| 3 | | 2→0 | | -2 | 3 | | 2 |
| 5 | | 2→0 | | -2 | 4 | | 2 |
| 6 | | 2→1 | | -1 | 4 | | 3 |
| 8 | | 2→0 | | -2 | 5 | | 2 |
| 12 | | 2→1 | | -1 | 6 | | 2 |
| 14 | | 0→2 | | 2 | 6 | | 4 |
| 15 | | 1→2 | | 1 | 6 | | 5 |
| 17 | | 2→0 | | -2 | 7 | | 2 |
| 18 | | 2→1 | | -1 | 7 | | 3 |
| 23 | | 2→1 | | -1 | 8 | | 2 |
| 27 | | 2→1 | | -1 | 8 | | 6 |
| 91 | | 2→1 | | -1 | 14 | | 13 |
| 104 | | 2→1 | | -1 | 15 | | 13 |
| *Anolis* | |  | |  |  | |  |
| 102 | | 2→1 | | -1 | 15 | | 11 |
| 103 | | 1/2→0 | | -1,5 | 15 | | 12 |
| 119 | | 2→1 | | -1 | 16 | | 14 |
| 134 | | 1→0 | | -1 | 17 | | 14 |
| *Iguana* | |  | |  |  | |  |
| 91 | | 2→0 | | -2 | 14 | | 13 |
| 133 | | 2→0 | | -2 | 17 | | 13 |
| 149 | | 2→0 | | -2 | 18 | | 13 |
| 152 | | 1/2→0 | | -1,5 | 18 | | 16 |
| *L. gravenhorsti* | |  | |  |  | |  |
| 25 | | 1→0 | | -1 | 8 | | 4 |
| 54 | | 2→0 | | -2 | 11 | | 9 |
| 55 | | 2→0 | | -2 | 11 | | 10 |
| 119 | | 2→1 | | -1 | 16 | | 14 |
| 120 | | 2→1 | | -1 | 16 | | 15 |
| 150 | | 2→1 | | -1 | 18 | | 14 |
| 151 | | 2→1 | | -1 | 18 | | 15 |
| *L. tenuis* | |  | |  |  | |  |
| 5 | | 2→1 | | -1 | 4 | | 2 |
| 14 | | 1→2 | | 1 | 6 | | 4 |
| 91 | | 2→1 | | -1 | 14 | | 13 |
| 132 | | 2→1 | | -1 | 17 | | 12 |
| 133 | | 2→0 | | -2 | 17 | | 13 |
| 190 | | 2→1 | | -1 | 20 | | 19 |
| *Strophurus* | |  | |  |  | |  |
| 104 | | 2→1 | | -1 | 15 | | 13 |
| 105 | | 1/2→0 | | -1,5 | 15 | | 14 |
| *Amalosia* | |  | |  |  | |  |
| 91 | | 2→1 | | -1 | 14 | | 13 |
| 135 | | 2→1 | | -1 | 17 | | 15 |
| 136 | | 1→2 | | 1 | 17 | | 16 |
| 151 | | 2→1 | | -1 | 18 | | 15 |
| 153 | | 2→1 | | -1 | 18 | | 17 |
| *Eublepharis* | |  | |  |  | |  |
| 91 | | 2→1 | | -1 | 14 | | 13 |
| 133 | | 2→1 | | -1 | 17 | | 13 |
| 135 | | 2→0 | | -2 | 17 | | 15 |
| *Gehyra* | |  | |  |  | |  |
| 55 | | 2→1 | | -1 | 11 | | 10 |
| 65 | | 2→1 | | -1 | 12 | | 10 |
| 104 | | 2→0 | | -2 | 15 | | 13 |
| 136 | | 1→0 | | -1 | 17 | | 16 |
| *Chondrodactylus* | |  | |  |  | |  |
| 119 | | 1/2→0 | | -1,5 | 16 | | 14 |
| 134 | | 0/1→2 | | 1,5 | 17 | | 14 |
| 136 | | 1→2 | | 1 | 17 | | 16 |
| 153 | | 2→1 | | -1 | 18 | | 17 |
| *Tarentola* | |  | |  |  | |  |
| 54 | | 2→1 | | -1 | 11 | | 9 |
| 65 | | 2→1 | | -1 | 12 | | 10 |
| 103 | | 2→0 | | -2 | 15 | | 12 |
| 132 | | 2→1 | | -1 | 17 | | 12 |
| 150 | | 2→0 | | -2 | 18 | | 14 |
| *Zootoca* | |  | |  |  | |  |
| 105 | | 1→2 | | 1 | 15 | | 14 |
| 120 | | 2→1 | | -1 | 16 | | 15 |
| 153 | | 2→1 | | -1 | 18 | | 17 |
| *Calyptommatus* | |  | |  |  | |  |
| 103 | | 2→1 | | -1 | 15 | | 12 |
| 133 | | 1→0 | | -1 | 17 | | 13 |
| 134 | | 1→0 | | -1 | 17 | | 14 |
| *Nothobachia* | |  | |  |  | |  |
| 66 | | 1→0 | | -1 | 12 | | 11 |
| 91 | | 2→1 | | -1 | 14 | | 13 |
| *Python* | |  | |  |  | |  |
| 104 | | 2→0 | | -2 | 15 | | 13 |
| 133 | | 1→0 | | -1 | 17 | | 13 |
| 169 | | 2→1 | | -1 | 19 | | 16 |
| 184 | | 2→1 | | -1 | 20 | | 13 |
| 187 | | 1/2→0 | | -1,5 | 20 | | 16 |
| 190 | | 2→0 | | -2 | 20 | | 19 |
| *Boaedon* | |  | |  |  | |  |
| 53 | | 2→1 | | -1 | 11 | | 8 |
| 99 | | 2→1 | | -1 | 15 | | 8 |
| 102 | | 2→1 | | -1 | 15 | | 11 |
| 104 | | 2→0 | | -2 | 15 | | 13 |
| 118 | | 2→1 | | -1 | 16 | | 13 |
| 133 | | 1→0 | | -1 | 17 | | 13 |
| 184 | | 2→1 | | -1 | 20 | | 13 |
| 187 | | 2→1 | | -1 | 20 | | 16 |
| 190 | | 2→0 | | -2 | 20 | | 19 |
| *Thamnophis* | |  | |  |  | |  |
| 3 | | 1/2→0 | | -1,5 | 3 | | 2 |
| 5 | | 2→0 | | -2 | 4 | | 2 |
| 13 | | 0/1→2 | | 1,5 | 6 | | 3 |
| 14 | | 1→2 | | 1 | 6 | | 4 |
| 17 | | 2→1 | | -1 | 7 | | 2 |
| 20 | | 1→0 | | -1 | 7 | | 5 |
| 21 | 2→0 | -2 | | 7 | 6 | |  |
| 44 | 2→1 | -1 | | 10 | 8 | |  |
| 135 | 2→0 | -2 | | 17 | 15 | |  |
| *Vipera* |  |  | |  |  | |  |
| 133 | 0/1→2 | 1,5 | | 17 | 13 | |  |
| 136 | 0→2 | 2 | | 17 | 16 | |  |
| *Varanus rosenbergi* |  |  | |  |  | |  |
| 118 | 2→1 | -1 | | 16 | 13 | |  |
| 119 | 2→0 | -2 | | 16 | 14 | |  |
| 120 | 2→0 | -2 | | 16 | 15 | |  |
| 136 | 1→2 | 1 | | 17 | 16 | |  |
| 150 | 2→1 | -1 | | 18 | 14 | |  |
| *Varanus panoptes* |  |  | |  |  | |  |
| 91 | 2→0 | -2 | | 14 | 13 | |  |
| 99 | 2→0 | -2 | | 15 | 8 | |  |
| 102 | 2→1 | -1 | | 15 | 11 | |  |
| 104 | 2→0 | -2 | | 15 | 13 | |  |
| 133 | 1/2→0 | -1,5 | | 17 | 13 | |  |
| 136 | 1→0 | -1 | | 17 | 16 | |  |
| 171 | 2→1 | -1 | | 19 | 18 | |  |
| *Varanus indicus* |  |  | |  |  | |  |
| 103 | 2→0 | -2 | | 15 | 12 | |  |
| 152 | 2→1 | -1 | | 18 | 16 | |  |
