# Supplementary material for: Evolution of developmental sequences in lepidosaurs
Source: PeerJ. 2017 Apr 27;5:e3262. doi: 10.7717/peerj.3262 (PMC5410152; doi:10.7717/peerj.3262)
Supplement: Supplemental Information 15 [file peerj-05-3262-s015.docx]

|  | **95% confidence interval** | |
| --- | --- | --- |
| **Taxon** | **Lower bound** | **Upper bound** |
| Molecular phylogeny, unordered characters | | |
| *Amalosia* | -1.4841 | 1.4841 |
| *Eublepharis* | -2.3229 | 2.3229 |
| *Gehyra* | -1.1940 | 1.1940 |
| *Tarentola* | -1.6019 | 1.6019 |
| *Python* | -4.3027 | 4.3027 |
| *Boaedon* | -1.4841 | 1.4841 |
| *Thamnophis* | -1.1636 | 1.1636 |
| *Varanus indicus* | -1.2417 | 1.2417 |
| *Varanus panoptes* | -1.5480 | 1.5480 |
| *Varanus rosenbergi* | -2.2992 | 2.2992 |
| *Pogona* | -1.6720 | 1.6720 |
| *Furcifer* | -1.3857 | 1.3857 |
| *Chamaeleo* | -2.1741 | 2.1741 |
| *Tropidurus* | -1.6258 | 1.6258 |
| *Iguana* | -2.9052 | 2.9052 |
| *Anolis* | -4.3027 | 4.3027 |
| *Liolaemus* | -4.3027 | 4.3027 |
| *L. gravenhorsti* | -0.4863 | 1.8197 |
| *L. tenuis* | -0.9414 | 0.9414 |
| *Mabuya* | -2.0989 | 2.0989 |
| Unidentata | -4.3027 | 4.3027 |
| Gymnophthalmidae | -1.7560 | 1.7560 |
| Molecular phylogeny, unordered characters | | |
| *Strophurus* | -5.4141 | 5.4141 |
| *Amalosia* | -1.6258 | 1.2658 |
| *Eublepharis* | -3.8977 | 3.8977 |
| *Gehyra* | -1.5103 | 1.5103 |
| *Chondrodactylus* | -4.0568 | 4.0568 |
| *Tarentola* | -2.2016 | 2.2016 |
| *Calyptommatus* | -1.7560 | 1.7560 |
| *Zootoca* | -2.5971 | 2.5971 |
| *Python* | -5.4141 | 5.4141 |
| *Boaedon* | -1.9632 | 1.9632 |
| *Thamnophis* | -1.5681 | 1.5681 |
| *Vipera* | -2.2751 | 2.2751 |
| *Varanus rosenbergi* | -2.6549 | 2.6549 |
| *Varanus panoptes* | -2.0246 | 2.0246 |
| *Pogona* | -1.6019 | 1.6019 |
| *Calotes* | -1.1682 | 1.1682 |
| *Agama* | -1.8477 | 1.8477 |
| *Furcifer* | -1.1831 | 1.1831 |
| *Chamaeleo* | -3.3284 | 3.3284 |
| *Uta* | -2.7415 | 2.7415 |
| *Tropidurus* | -3.1656 | 3.1656 |
| *Iguana* | -5.3961 | 5.3961 |
| *L. gravenhorsti* | -1.6173 | 1.6173 |
| *L. tenuis* | -1.1940 | 1.1940 |
| *Mabuya* | -1.9217 | 1.9217 |
| Unidentata | -5.4141 | 5.4141 |
| Gymnophthalmidae | -1.7095 | 1.7095 |
| Toxicofera | -4.3027 | 4.3027 |
| Serpentes | -5.4141 | 5.4141 |
| *Thamnophis* + *Vipera* | -5.4141 | 5.4141 |
| *Agama* + *Calotes* | -3.1824 | 3.1824 |
| Morphological phylogeny, unordered characters | | |
| *Iguana* | -2.2403 | 2.2403 |
| *Tropidurus* | -4.3027 | 4.3027 |
| *Pogona* | -1.4131 | 1.4131 |
| *Furcifer* | -1.3857 | 1.3857 |
| *Chamaeleo* | -2.0512 | 2.0512 |
| *Anolis* | -1.7560 | 1.7560 |
| *Liolaemus* | -1.5480 | 1.5480 |
| *L. gravenhorsti* | -1.3857 | 1.3857 |
| *L. tenuis* | -1.2154 | 1.2154 |
| *Amalosia* | -1.4841 | 1.4841 |
| *Eublepharis* | -1.9632 | 1.9632 |
| *Gehyra* | -1.7560 | 1.7560 |
| *Tarentola* | -1.6019 | 1.6019 |
| *Python* | -2.6340 | 2.6340 |
| *Boaedon* | -2.1358 | 2.1358 |
| *Thamnophis* | -1.2747 | 1.2747 |
| *Varanus panoptes* | -1.6019 | 1.6019 |
| *Varanus rosenbergi* | -2.4833 | 2.4833 |
| Scincomorpha | -4.3027 | 4.3027 |
| *Mabuya* | -2.9120 | 2.9120 |
| Gymnophthalmidae | -1.7560 | 1.7560 |
| Morphological phylogeny, ordered characters | | |
| *Calotes* | -1.1184 | 1.1184 |
| *Pogona* | -1.4821 | 1.4821 |
| *Agama* | -5.4141 | 5.4141 |
| *Furcifer* | -1.1682 | 1.1682 |
| *Chamaeleo* | -3.0350 | 3.0350 |
| *Anolis* | -1.8477 | 1.8477 |
| *Iguana* | -4.7485 | 4.7485 |
| *Liolaemus* | -2.2809 | 2.2809 |
| *L. gravenhorsti* | -1.7612 | 1.7612 |
| *L. tenuis* | -1.4380 | 1.4380 |
| *Strophurus* | -5.4141 | 5.4141 |
| *Amalosia* | -1.6258 | 1.6258 |
| *Eublepharis* | -3.8977 | 3.8977 |
| *Gehyra* | -1.5103 | 1.5103 |
| *Chondrodactylus* | -4.0568 | 4.0568 |
| *Tarentola* | -1.4131 | 1.4131 |
| *Zootoca* | -1.7560 | 1.7560 |
| *Calyptommatus* | -1.7560 | 1.7560 |
| *Python* | -3.2684 | 3.2684 |
| *Boaedon* | -2.5671 | 2.5671 |
| *Thamnophis* | -2.2049 | 2.2049 |
| *Vipera* | -7.5552 | 7.5552 |
| *Varanus panoptes* | -2.3299 | 2.3299 |
| *Varanus rosenbergi* | -3.3843 | 3.3843 |
| *Mabuya* | -3.2224 | 3.2224 |
| Gymnophthalmidae | -2.2654 | 2.2654 |
| Scleroglossa | -4.3027 | 4.3027 |
| Pleurodonta | -6.5724 | 6.5724 |
